# Monthly Summary

**Published:** 1888-04

**Authors:** 


### Monthly Summary.

---

A CHEAP MATRIX.—Recently I have been successful with a matrix which I think will be of value to dentists who do not make gold crowns, and for patients who cannot pay for them, but who are willing to pay a moderate fee for saving badly decayed and broken molars. It is made from the composition silver strips that come for polishing between the teeth. One of the proper width is selected and cut a little longer than the circumference of the tooth to be treated. It is bent around it with the fingers and the ends punched together with a pair of pliers, drawing it tight. It is then removed and placed on a mandril. My mandril is a round steel 7 in. long, 5-8 in. diameter at the large end, and 1-8 in. at the small end.

The ends of the band are folded over similar to the way a stove-pipe seam is made. If the fold is not strong enough to hold, dip a piece of No. 60 tinfoil in soldering fluid, lay it on the seam, and hold it in the flame of an alcohol lamp. It will be soldered. Adjust the band on the tooth. If it fits, have the patient close his mouth to see that the articulation is all right. Now if the tooth is all prepared fill with amalgam. When full take a wad of cotton and press the filling with it—using your thumb. It will make the filling very compact, and bring the surplus mercury to the surface. Do not remove the band, but instruct the patient to call the next day or week and have it taken off. What I claim for this matrix is, it can be left on the tooth till the filling is hard. It is not expensive or hard to make. It is unequaled in filling large cavities with thin, frail walls by grinding down the walls and building the filling up over the edge, making the whole grinding surface of the tooth amalgam.—*W. Sloan*, *Peoria*, *Ill.*

---

LEAD OR AMALGAM INSTEAD OF GOLD.—We know that teeth that have in ancient days been filled with lead have been well preserved; and that lead foil was particularly recommended for filling teeth more than sixty years ago. Perhaps it was not for this reason that its use was advocated, but we know that lead and its products are exceedingly sedative in thier influence on sensitive teeth. Tin comes next to it, with the same qualities, at least in degree. We know that amalgam will often be successful where gold would fail. I can recall many cases in my practice where I have filled large molars again and again with gold only to find each time that the dentine would dissolve about the margins of the fillings, and ultimately I would be obliged to remove and refill with amalgam; the decay would then be entirely arrested, and the operations have been successful to this day. My theory was that the amalgam threw out a species of oxidation from its surface coming in contact with the fluids of the mouth, so that a sort of galvanic process was set up, which in time resulted in fossilizing the tooth-substance. In some cases where I have left a portion of the soft, partly decomposed dentine in the cavity, years afterward, when I removed the filling, I have found that dentine not only fossilized and recalcified, but became hard and crepitous like glass.—*Dr. Dwinelle, New York.*

---

HOW TO CUT A BOTTLE.—A correspondent of the *Chem. and Drug.*, in describing how to make a percolator, mentions the following method of cutting a bottle: I was first shown how to do it by an ingenious mechanic, and have since seen the same published. Put the bottle on a level foundation and fill up with oil (I use linseed oil, being able to use it in paint afterwards) as far as you wish the line of separation to be. Next get a rod of iron as large as possible, but small enough to go into the mouth of the bottle. Make the iron almost white hot and dip it into the oil. In a very short time a crack will be heard when the iron can be taken out, and the bottle will be found as neatly cut as if with a diamond. Should the bottle be very thick, and the crack not heard in a minute or so, a dash of cold water outside will settle the business.

---

WATER AT MEALS.—Opinions differ as to the effect of the free ingestion of water at meal times, but the view most generally received is that it dilutes the gastric juice and so retards digestion. Apart from the fact that a moderate delay in the process is by no means a disadvantage, as Sir William Roberts has shown in his explanation of the popularity of tea and coffee, it is more than doubtful whether any such effect is in reality produced. When ingested during meals water may do good by washing out the digested food and by exposing the undigested part more thoroughly to the action of the digestive ferments. Pepsin is a catalyptic body, and a given quantity will work almost indefinitely, provided the peptones are removed as they are formed. The good effects of water, drank freely before meals, has, howover, another beneficial result—it washes away the mucus which is secreted by the mucous membrane during intervals of repose, and favors peristalsis of the whole alimentary tract. The membrane thus cleaned is in a much better condition to receive food and convert it into soluble compounds.—*Med. Reg.*

---

USES OF CHLORA-PERCHA.—There are many pat uses to which chloro-percha can be put, which I am satisfied the profession do not recognize, to wit: For securing arsenical applications in or on shallow surfaces; for instantly sealing accidental punctures of the rubber dams *in situ*, a small piece of punk dipt in chloro-percha and laid on the defect, for covering plastic fillings under the hardening process, and many other uses which suggest themselves to a bright, practical mind. Glycerin also has many useful qualities for coating cavities and approaches to root-canals which are to be filled with chloro-percha (the latter will not stick on a surface previously covered with glycerin.) For covering all glass stoppers to prevent sticking, for coating (with only a trace) instruments used in working the plastics used on burnishers and stone in place of oil, etc.—*G. A. Bowman, in Dental Review.*

---

IRREGULARITIES.—At the last American Dental Association, Dr. W. S. Barrett presented the case of a miss of fourteen, whose anterior teeth projected and were much shortened as if from thumb sucking; which, however, was not the cause, the difficulty in all probability arising from the premature extraction of teeth. There were but two molars in the upper jaw, and the problem was to draw the six anterior teeth back by means of six posterior ones without danger of moving the latter. It was accomplished by carefully banding the bicuspids and molar on each side, as if for gold crowns, and then attaching these bands together by soldering to them a side plate which carried guides and a screw for drawing back a thin plate or strap, which passed across the faces of the anterior teeth, the whole apparatus being made of gold. The banding of the plate presented so immovable an anchorage that tipping of the teeth was impossible.—*Items of Interest.*

---

A PHYSICIAN AND THE TOOTHACHE.—Dr. Peete relates the following incident in the *Archives*: "This man came to me," he said, "with a severe pain in one of his lower jaw teeth, which was somewhat decayed. I told him, *of course*, the nerve of the tooth must be destroyed, the first thing. I took a vial containing, perhaps, an ounce and a half of nitric acid, and ordered the patient to keep perfectly quiet, while I poured a drop of the acid into the cavity.

I had confidence in my steadiness of hand, and knew that I could pour out a single drop without any difficulty. Just as I had the neck of the bottle close enough to the tooth, the man made a sudden movement, and at least an ounce of acid was spilled in his mouth. But I seldom lose presence of mind, and promptly seized a handful of carbonate of potash and crammed it into his mouth. No material damage was done, and the tooth has never ached since."

---

THE MOST CONVENIENT MATRIX is one made of copper. Even where the cavity extends under the gums, take a piece of soft copper which you can make very soft by annealing, cut and adapt to your tooth, then remove and solder, either with soft solder or silver solder, then put on your dam and dry your tooth; mix phosphate or use chloroform and gutta-percha on the edge of your band or matrix toward the gums, if you have not the band as tight as you wish, use a wedge between the band and tooth. Dip the wedge in the gutta-percha solution to make it stick. When you have everything dry, you can burnish out your band so as to contour as much as you like. I use copper as patterns for all crowns as it is soft and easily adapted to the root or tooth, then I cut the gold by my pattern and solder it.—*J. S. Marsh, Chicago, in Items of Interest.*

